# Pharmaceutical Effects of Inhibiting the Soluble Epoxide Hydrolase in Canine Osteoarthritis

**DOI:** 10.3389/fphar.2019.00533

**Published:** 2019-05-31

**Authors:** Cindy B. McReynolds, Sung Hee Hwang, Jun Yang, Debin Wan, Karen Wagner, Christophe Morisseau, Dongyang Li, William K. Schmidt, Bruce D. Hammock

**Affiliations:** ^1^Department of Entomology and Nematology, UC Davis Comprehensive Cancer Center, University of California, Davis, Davis, CA, United States; ^2^EicOsis, Davis, CA, United States

**Keywords:** soluble epoxide hydrolase, osteoarthritis, epoxyeicostrienoic acids, analgesic, non-opioid analgesic, non-NSAID analgesic

## Abstract

Osteoarthritis (OA) is a degenerative joint disease that causes pain and bone deterioration driven by an increase in prostaglandins (PGs) and inflammatory cytokines. Current treatments focus on inhibiting prostaglandin production, a pro-inflammatory lipid metabolite, with NSAID drugs; however, other lipid signaling targets could provide safer and more effective treatment strategies. Epoxides of polyunsaturated fatty acids (PUFAs) are anti-inflammatory lipid mediators that are rapidly metabolized by the soluble epoxide hydrolase (sEH) into corresponding vicinal diols. Interestingly, diol levels are increased in the synovial fluid of humans with OA, warranting further research on the biological role of this lipid pathway in the progression of OA. sEH inhibitors (sEHI) stabilize these biologically active, anti-inflammatory lipid epoxides, resulting in analgesia in both neuropathic, and inflammatory pain conditions. Most experimental studies testing the analgesic effects of sEH inhibitors have used experimental rodent models, which do not completely represent the complex etiology of painful diseases. Here, we tested the efficacy of sEHI in aged dogs with natural arthritis to provide a better representation of the clinical manifestations of pain. Two sEHI were administered orally, once daily for 5 days to dogs with naturally occurring arthritis to assess efficacy and pharmacokinetics. Blinded technicians recorded the behavior of the arthritic dogs based on pre-determined criteria to assess pain and function. After 5 days, EC1728 significantly reduced pain at a dose of 5 mg/kg compared to vehicle controls. Pharmacokinetic evaluation showed concentrations exceeding the enzyme potency in both plasma and synovial fluid. *In vitro* data showed that epoxyeicosatrienoic acid (EETs), epoxide metabolites of arachidonic acid, decreased inflammatory cytokines, IL-6 and TNF-α, and reduced cytotoxicity in canine chondrocytes challenged with IL1β to simulate an arthritic environment. These results provide the first example of altering lipid epoxides as a therapeutic target for OA potentially acting by protecting chondrocytes from inflammatory induced cytotoxicity. Considering the challenges and high variability of naturally occurring disease in aged dogs, these data provide initial proof of concept justification that inhibiting the sEH is a non-NSAID, non-opioid, disease altering strategy for treating OA, and warrants further investigation.

## Introduction

Arthritis is a collective term used to describe joint disease and pain; however, there are many types of arthritis with very different etiologies. The two most common forms of arthritis are rheumatoid arthritis (RA), an autoimmune disease, and osteoarthritis (OA), a degenerative disease that results in breakdown of cartilage between bone leading to joint pain, swelling, reduced mobility, and bone degradation. Cartilage is comprised of chondrocyte cells that are responsible for generating the extracellular matrix and maintaining healthy cartilage tissue. Upon injury or disease, inflammatory cytokines lead to chondrocyte cytotoxicity and if not resolved, cartilage destruction. Recent medical advances for RA target immune suppression through monoclonal antibodies against anti-inflammatory targets. These treatments have shown promise in effectively treating RA (Tanaka et al., 2014); but OA, despite also having a common inflammatory cause, remains largely untreated, and patients often turn to NSAIDs and opioids to alleviate pain with no benefit to treating or stopping the progression of the disease (Ivers et al., 2012). In addition, these treatment options have serious side effects. For example, over 100,000 people are hospitalized each year for NSAID complications, and addiction to opioids has become a national health crisis affecting over 2 million people. Considering these serious side effects, safer, and more effective options are needed for patients suffering from OA.

**FIGURE 1:**
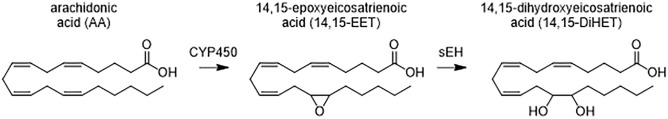
Metabolism of arachidonic acid (AA) by cytochrome P450 (CYP450) generates anti-inflammatory fatty acid epoxides (EETs) that are degraded by the soluble epoxide hydrolase (sEH) to the corresponding 1,2 diols (DiHETs). DiHETs are more polar and diffuse out of the cells and are readily conjugated and excreted. The omega-6 fatty acid, AA, and representative metabolites on the 14, 15 carbon position are shown. Similar epoxides and vicinal diols are formed on the double bonds, and other omega-3 and 6 PUFAs are metabolized in a similar fashion.

Osteoarthritis is characterized by an increase in both inflammatory cytokines and inflammatory metabolites of arachidonic acid (AA) (Valdes et al., 2018). AA is an omega-6 polyunsaturated fatty acid (PUFA) that is metabolized primarily by three main enzyme systems: cyclooxygenases (COX), lipoxygenases, and CYP450s. COX metabolism of AA produces proinflammatory prostaglandins (PGs) that increase inflammatory cytokines and drive the progression of OA. Blocking PG production with NSAIDs that inhibit COX function has been an important approach to treating the disease (da Costa et al., 2017). However, despite the prevalent use of NSAIDs prescribed to OA patients, the efficacy and side-effects vary widely among patients, and long-term NSAID use is associated with increased risk of life-threatening toxicities (Marcum and Hanlon, 2010). Targeting other lipid mediators, such as the CYP450 branch of the AA cascade instead of the COX branch may provide better treatment alternatives. CYP450 epoxide metabolites of AA, or epoxyeicosatrienoic acid (EETs) prevent the translocation of Nf-κB into the nucleus thereby resulting in decreased inflammatory cytokines (Node et al., 1999). Additionally, EETs reduce ER-stress to help maintain homeostasis and have been effective in reducing neuropathic pain (Inceoglu et al., 2017). For these reasons, EETs are viewed as largely anti-inflammatory and beneficial compounds, but are often limited in efficacy due to their rapid degradation by the sEH into diol metabolites for excretion (Shimizu, 2009; Figure 1). Recently, the sEH products of EET metabolism [specifically 11,12 and 14,15 dihydroxyeicosatrienoic acid (DiHET)] were found in higher concentrations in arthritic joints compared to healthy or unaffected joints (Valdes et al., 2018). Chemical inhibitors of sEH increase the concentration of EETs and other epoxides of omega-3 and -6 fatty acids in the body by decreasing the formation of corresponding diol metabolites (Morisseau and Hammock, 2013). Increasing epoxy-fatty acid (EpFA) concentration through inhibition of the sEH resolves a variety of disease states in laboratory settings, and efforts are ongoing to evaluate inhibition of sEH as an effective treatment for pain in clinical applications (Wagner et al., 2017b).

The sEH inhibitor, EC1728 (*trans*-4-{4-[3-(4-trifluoromethoxy-phenyl)-ureido]-cyclohexyloxy}-benzoic acid, also referred to as *t*-TUCB), is a potent sEHI that has shown efficacy in several animal models (Wagner et al., 2017a), and in treating a natural, neuropathic pain condition in horses called laminitis (Guedes et al., 2017). Although EC1728 has been effective in multiple models of pain, it has poor physical properties, such as high melting point (212.2°C) and low water solubility (5 mg/L in PBS), that limits its formulation, and bioavailability. Efforts were made to develop new sEHI with improved physiochemical properties. Because optimization of sEH inhibitors demonstrated that carbamates and amides are generally less active than urea pharmacophores (Kodani et al., 2018), new compounds focused on keeping the urea pharmacophore. Attempts at improving the physicochemical characteristics of EC1728 identified EC3039 (*trans*-4-{4-[3-methyl-3-(4-trifluoromethoxy-phenyl)-ureido]-cyclohexyloxy}-benzoic acid, also referred to as t-MTUCB) as a structurally similar compound that differs only in the addition of a N-methyl group to the urea pharmacophore (Table 1). The results below describe the evaluation of these two sEHI in a preclinical, randomized, and blinded study for their ability to alleviate pain in dogs with natural OA. *In vitro* studies tested the hypothesis that sEH inhibition reduces pain by increasing EETs that decrease inflammation in the chondrocytes.

**Table 1:** EC1728 and EC3039 are potent sEH inhibitors.

Structure	Mol. Wt. (g/mol)	Melting point (°C)	Cal. LogP^1^	Canine sEH IC_50_ (ng/mL)^2^	Solubility (mg/mL)^3^	
					PBS pH 7.4	PEG300
EC1728 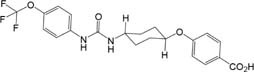	438.40	240–244	5.0	0.4 ± 0.1	0.005 ± 0.001	97 ± 2
EC3039 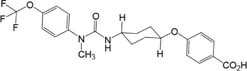	452.43	92.4–94.7	5.0	2.0 ± 0.3	0.130 ± 0.015	99 ± 1

*EC3039 has a lower melting point and higher solubility but is less potent inhibiting the canine sEH enzyme compared to EC1728. ^1^LogP was calculated using ChemDraw Professional version 18.0.0.231. ^2^IC_50_ was measured using PHOME [(S) = 50 μM] as fluorescent substrate with partially purified canine sEH from dog liver (Wolf et al., 2006). The residual esterase activity was completely inhibited with paraoxon (100 μM) in assay buffer. ^3^Solubility was measured at 30°C as described (Hwang et al., 2007).*

## Materials and Methods

### sEHI (EC1728 and EC3039)

EC1728 was synthesized in-house as previously described (Hwang et al., 2007), synthesis of EC3039 and deuterated 1728 *trans*-4-{4-[3-(4-trifluoromethoxy-phenyl)-ureido]-cyclo-hexyloxy}-benzoic-2,3,5,6-*d*_4_ acid, also referred to as 3049, *t*-TUCB-*d*_4_) is described in the Supplementary Material.

IC_50_ was determined as previously described (Wolf et al., 2006). Briefly, canine enzyme was partially purified on a Q-sepharose column from liver cytosol (∼10-fold purification) as described in (Tsai et al., 2010). The residual esterase activity was completely inhibited with paraoxon purchased from Chem Services Inc. (West Chester, PA, catalog number 42417404) at a concentration of 100 μM in assay buffer.

#### EET Mixture

Epoxyeicosatrienoic acid (5,6 EET, 8,9 EET, 10,11 EET, and 13,14 EET) were purchased from Cayman Chemicals (catalog numbers 50211, 50351, 50511, and 50651) and diluted 1000-fold for a final concentration of 0.1 μg/mL for each EET (0.4 μg/mL total). Meloxicam iv injectable was purchased from the UC Davis veterinary pharmacy at 5 mg/mL. Stock solutions were diluted to 1 mg/mL in ethanol and added to cell cultures at a final concentration of 1 μg/mL and 0.1% ethanol.

#### Solubility of sEHI

Solubility was determined by shaking the compounds in phosphate buffered saline (pH 7.4) at 40°C for 24 h in glass tubes. The non-dissolved compounds were filtered through a 0.22 μm centrifuge filter at 40°C and the supernatant was further diluted 10 times with methanol. The samples were analyzed by LC/MS-MS.

### HPLC/MS-MS Method

Concentrations of EC3039 and EC1728 were determined by LC/MS/MS analysis as previously described (Tsai et al., 2010) and defined in detail in the Section Supplementary Material “LC/MS/MS Method for PK Analyses of 1728 and 3039” and optimized parameters listed in Supplementary Table S1.

### *In vitro* Model of Chondrocyte Cytotoxicity

#### Cell Culture

Canine chondrocytes (CnC) were purchased from Cell Applications, Inc. (catalog number Cn402-05). Cells arrived at Passage 1 and were cultured in complete growth media (Canine Chondrocyte Growth Medium supplemented with 10% Canine Chondrocyte Growth Supplement (CGS), both from Cell Applications, Inc.) and maintained at 37°C in a 5% CO_2_ atmosphere. Once cells reached 80% confluence, they were passaged by removing the media, washing with PBS (warmed to room temperature) before adding 0.05% Trypsin-EDTA (Gibco Life Technologies, catalog number 25300) also warmed to room temperature. Cells were kept at room temperature and monitored for detachment (∼2 min). Detached cells were added to 3× volume of full growth media and centrifuged to remove trypsin. Cells were reseeded at a concentration of 15,000 cells/cm^2^ to reach 80% confluence in 3 days. Cells were expanded and frozen at the third passage in CGS containing 10% DMSO. For individual experiments, fresh cells were thawed and used at passage 5.

#### Chondrocyte Cytotoxicity *in vitro*

Canine chondrocytes cells were plated in 96-well plates at a density of 15,000 cells/cm^2^. Optimal cell number was predetermined by a cell seeding experiment outlined in O’Brien et al. (2000) Cells were treated with recombinant canine IL1β (Novus Biologicals catalog number 3747-CL-025) in CGS-free media for 2 h at 37°C. After 2 h, the IL1β treated media was aspirated and replaced with complete growth media supplemented with either vehicle (0.1% ethanol), regioisomers of EETs, or meloxicam for 48-h before assessing inflammatory cytokines and cytotoxicity. Cells were also pretreated with meloxicam or EET mixture 30 min prior to adding IL1β treatment for 2-h. After IL1β was removed, treatments were added to complete growth media for 48-h as described above. Treatment conditions are described in the Section “Results.”

#### Cytotoxicity Assay

After the CnC cells were treated for 48-h under conditions described above, the media was removed and replaced with complete media containing 10% Alamar Blue (Life Technologies, catalog number DAL1025). The Alamar Blue assay relies on the metabolism of non-fluorescent resazurin to the highly fluorescent resorufin by healthy cells. Fluorescent intensity can be used to track cytotoxicity after xenobiotic treatments (Larson et al., 1997). After the addition of Alamar Blue, plates were protected from light and incubated at 30°C in a 5% CO_2_ atmosphere for 8 h. The conversion of resazurin was monitored by reading fluorescence at 570 excitations and 585 emission on a plate reader (Tecan M1000 Pro) and cytotoxicity was calculated as the ratio of fluorescence from treated cells to non-treated controls.

#### ELISA Determination of IL-6 and TNF-α

ELISA determination of IL-6 and TNF-α: DuoSet ELISA kits (R&D Systems, **RRID:SCR\_006140**) were used to quantify inflammatory cytokines, IL-6 (catalog number DY1609) and TNF-α (catalog number CATA00) in the media collected after 48-h of EET or vehicle treatments. Manufacturer’s instructions were followed with adaption to improve the assay sensitivity using polymeric horseradish peroxidase (PolyHRP) according to previous work (Li et al., 2017). The following adaptions were conducted: the original two separate steps of adding standards∖sample and biotinylated detection antibody with each incubated 2 h were changed to one-step simultaneous addition of both reagents with incubation time shortened to 1 h. After washing, the addition of streptavidin conjugated to horseradish peroxidase (Streptavidin-HRP) from the kit was replaced with streptavidin PolyHRP40 conjugate (25 ng/mL, 30 min) from SDT GmbH (Baesweiler, Germany). All incubations above were performed at room temperature with shaking (600 rpm, MTS 2/4 digital microtiter shaker, IKA, Germany).

### Microsomal Stability

Male and female dog liver microsomes (Xenotech) were diluted in potassium phosphate buffer (0.1M, pH 7.4), MgCl2 (3.15 mM), and sodium EDTA (1.05 mM) to a final protein concentration of 1 mg/mL. Compounds were added at 0.1 mM in methanol at 1% and microsomes were activated with 5% (v/v) NADPH regenerating system (100 μL sodium phosphate buffer (0.1M, pH 7.4), 50 μL, glucose-6-phosphate in sodium phosphate buffer (0.1M, pH 7.4), 50 μL NADP+ in sodium phosphate (0.1M, pH 7.4), and 50 μL of 100 Units/mL of glucose-6-dehydrogenase). Compounds were incubated for 60 min at 37°C and quenched with 4× volume of ice-cold methanol containing deuterated EC1728 as an internal standard. Compounds were incubated without microsomes or NADPH as negative controls.

### Pain Assessment in Arthritic Dogs

#### *In vivo* Studies

Studies were contracted with InterVivo Solutions, Toronto, ON, Canada to assess pain and pharmacokinetics of E1728 and EC3039 in aged beagle dogs with OA. This study was conducted in accordance with InterVivo’s approved IACUC protocol, in compliance with the Animal Welfare Act (AWA) and Public Health Service Policy on Humane Care and Use of Laboratory Animals.

#### Drug Administrations

There were five groups in total (placebo, 1 mg/kg EC1728, 5 mg/kg EC1728, 1 mg/kg EC3039, and 5 mg/kg EC3039). The placebo group contained 8 osteoarthritic dogs. The four drug-treated groups contained 8 osteoarthritic dogs and two satellite dogs (1 male and 1 female) of similar age and weight for PK blood sampling. Time 0 served as the pretreatment control. A total of 48 animals were on study.

#### PK Analysis

Eight satellite dogs (2 per each group) had whole blood collected for PK analysis on study Days 0 through 4. On Day 1, blood was collected immediately prior to dosing, and again at 0.25, 0.5, 1, 2, 4, 6, and 8 (±5 min) after dosing. On Day 2 through 4, blood was collected 0.5 h (±5 min) after dosing. On Day 5 blood was collected immediately prior to dosing, and again at 0.25, 0.5, 1, 2, 4, 6, 8 (±5 min), 24, 48, and 72 h (±30 min) post treatment. The exact sampling time was noted in the study file. For all blood samples, a minimum of 3 mL of whole blood was collected from a suitable vein as per written and approved standard operating procedures. The blood was transferred to K_2_EDTA (spray dried) blood tube and inverted gently multiple times to ensure proper mixing of the blood and anticoagulant. On study Day 5, synovial fluid was collected by arthrocentesis immediately after the subject completed the questionnaire (approximately 3 h after dosing). Subjects were anesthetized as per written and approved Standard Operating Procedures. A minimum of 0.1 mL of synovial fluid was collected from each subject. Samples were frozen in an upright position and stored at -80°C ± 4°C before being shipped to UC Davis for analysis.

For PK analysis in the satellite group, individual parameters were calculated by fitting blood concentrations to a non-compartmental analysis using Kinetica software (Thermo Fisher Scientific version 5.1). Using the log-linear trapezoidal method, the area under the curve were calculated and were extrapolated to infinity using the last measured plasma concentration (C_last_), defined as the timepoint collected 72 h after the last dose or 8 hr after the first dose on day 1, divided by the terminal slope (λz).

#### Efficacy

The study was conducted as a blinded, parallel matched-group design. Dogs were randomized into groups based on pretreatment pain scores and each group was randomly assigned a treatment. Prior to randomization, each dog was assessed for arthritis by radiograph to assess the severity of OA by presence of osteophytes, subchondral sclerosis or joint effusion each scored on a scale of 0 (least) to 3 (worst) for each of 12 joints. Severity of the radiograph score was included in the randomization protocol and distributed between the groups, and there were no statistical differences between the averages of each group. Individual radiograph scores can be found in Supplementary Table S2. Treatment groups consisted of vehicle control, 1 mg/kg EC3039, 5 mg/kg EC3039, 1 mg/kg EC1728, and 5 mg/kg EC1728. Compounds were dissolved in PEG300 to a concentration of 2.4 mg/mL (1 mg/kg) and 12 mg/mL (5 mg/kg) and administered as approximately 5 mL in size 12 gelatin capsules (Torpac) administered once daily on days 1 through 5. Vehicle group consisted of 5 mL PEG300 in capsule. Concurrent medications were not allowed and not needed during the study.

#### Pain and Function Assessments

Pain and function were assessed by a questionnaire answered by blinded technicians based on observation of study animals housed at InterVivo. The survey was adapted from the canine brief pain inventory (CBPI), which was developed based on clinical questionnaires in which owners score the function and pain level of their pets with existing pain conditions (Brown et al., 2007). Modifications to the questionnaire attempted to account for differences between owner pain evaluation of pets and pain evaluation of laboratory dogs by technical staff. For survey administration, blinded technicians attempted to elicit objective behavior-specific measures of function and pain level across a variety of normal canine behaviors (e.g., walking, trotting, galloping, rearing, and stair-climbing, etc.). For this, one technician was responsible for soliciting the relevant behaviors from a dog using encouragement and/or food rewards, while the evaluating technician scored function and observable pain using the modified CBPI questionnaire. Subjects were tested daily on Days -7 through -3, to develop baseline scores, and again on Days 1 through 5 administered 1.5 h after dosing. Dosing occurred in the morning, and observations were recorded 1.5 h after dosing, to limit time-of-day variability. Questions are listed in Supplementary Table S3. Animals in treatment groups had blood and synovial fluid collected ∼2 h after the last dose on day 5 (immediately following the study questionnaire) to confirm plasma concentrations in treated animals. Full pharmacokinetic assessments were collected from a satellite group of animals so as not to interfere with pain assessment.

#### Statistical Analysis

Canine pain and function data were analyzed using ANOVA, linear mixed effects model, with Tukey adjustment in R and repeated measures ANOVA. Correlations coefficients were calculated in Graphpad Prism using the non-parametric Spearman correlation coefficient. *In vitro* experiments were analyzed using ANOVA in Graphpad Prism. Data are reported significant if *p* < 0.05.

## Results

### sEHI Physicochemical Characteristics

EC3039 was synthesized and designed to have favorable physiochemical properties relative to EC1728. As crystals or larger particles, lipophilic urea compounds have poor oral availability. Also, Chu and Yalkowsky (2009) showed an association between lower melting point and increased absorption. Thus, improving the aqueous solubility and lowering the melting point were prioritized to improve the potential for efficacy in future *in vivo* studies. EC3039 had improved solubility and lower melting point compared to EC1728 but had a slightly less potent IC_50_ (5-times less potent than EC1728, Table 1). To assess the metabolic stability, both compounds were tested for stability after incubation in dog liver microsomes. Both compounds were relatively stable with EC1728 having over 95% remaining, and EC3039 having over 90% remaining in male liver microsomes and 85% remaining in female liver microsomes after 60-min incubations (Table 2).

**Table 2:** EC3039 and EC1728 are stable after 60-minute incubations with male and female dog liver microsomes.

Compound		% Parent	
		+ NADPH	− NADPH
EC3039^∗^	Males	90 ± 7	97 ± 4
	Females	85 ± 2	93 ± 5
EC1728	Males	103 ± 6	108 ± 8
	Females	98 ± 6	106 ± 9

*^∗^A small amount of EC1728 higher than the LOD (0.3 ng/mL) but lower than the LOQ (3 ng/ml) was detected in microsomes incubated with EC3039.*

### *In vivo* Pain Evaluation

To evaluate the PK of both EC1728 and EC3039 and the ability to alleviate pain in dogs with natural arthritis, two dose levels of EC1728 and EC3039 were administered orally for five consecutive days to aged dogs with arthritis.

### PK Analysis

**FIGURE 2:**
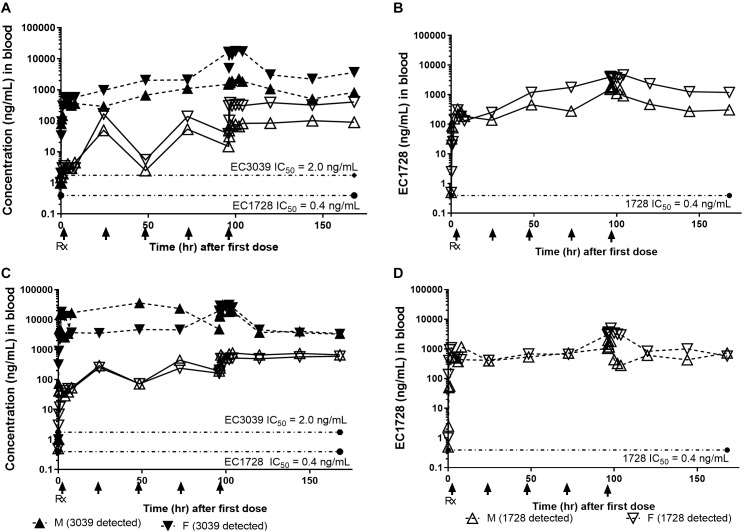
Concentrations of EC3039 and EC1728 in the blood exceeded the IC_50_ for sEH for all treatment groups for the duration of the study. The PK profile of animals treated with EC3039 **(A,C)** or EC1728 **(B,D)** in a satellite group of *n* = 2 dogs administered 1 mg/kg or 5 mg/kg orally once daily for 5 consecutive days is shown. Blood was collected at 0.25, 0.5, 1, 2, 4, 6, and 8 h after dosing on Day 1, and again on days 2 through 4 at 0.5 h after dosing. On Day 4, blood was collected immediately prior to dosing, and again at 0.25, 0.5, 1, 2, 4, 6, 8 (±5 min), 24, 48, and 72 h (±30 min) post treatment. Concentrations of EC1728 and EC3039 were well above the EC_50_ for the study duration. Blood concentrations increased over the 5 days of dosing in all treatment groups, and T_1/2_ calculated from the parent concentration predicts that steady-state is reached by day 5 for EC1728 and between days 5–8 for EC3039. Dogs administered EC3039 at 1 mg/kg **(A)** and 5 mg/kg **(C)** had detectable amounts of EC1728 in their blood presumably from demethylation of the urea by CYP450. The amount was proportional to EC3039 levels and above the EC_50_ for sEH, although approximately 3× lower than the dogs in the EC1728 treatment groups. A model could not be fitted for EC1728 detected from dogs administered EC3039 and T_1/2_ were not calculated. Dogs administered EC1728 at either 1 mg/kg **(B)** and 5 mg/kg **(D)** had no detectable amounts of EC3039 in their blood. Drug concentrations did not show a clear dose response for dogs treated with EC1728, and dogs treated with 1 mg/kg of EC1728 **(B)** had approximately half the AUC as dogs treated with 5 mg/kg EC1728 **(D)**.

In the satellite PK group (*n* = 2, 1 male and 1 female), concentrations of EC3039 and EC1728 increased through day 5 (Figure 2). Based on the reported half-life of both compounds (24–40 h, Table 3), it is expected that concentrations reported on day 5 represent the steady state values (Ito, 2011). Systemic exposure (C_max_ and AUC) increased in a more than dose proportional manner for EC3039 in both the male and female satellite PK dogs. On day 1, a fivefold increase in dose resulted in a 30 to 40-fold increase in C_max_ and approximate 18-fold increase in AUC for EC3039 (Figure 2A,B). Alternatively, a fivefold increase in dose for EC1728 resulted in a less than dose dependent increase in systemic exposure (3.25-fold increase in both C_max_ and AUC after fivefold dose increase) (Figure 2C,D). As the compounds reached steady-state, dose proportionality for EC1728 was less pronounced, with EC1728 having similar C_max_ and AUC for both doses (Table 3). This is likely due to the poor solubility of EC1728 where intestinal absorption is dependent upon the amount of drug that is in a true solution. A small portion (<10%) of 3039 was metabolized by demethylation to form EC1728 (Figure 2A,B).

**Table 3:** PK parameters of EC3039 and EC1728 administered to a satellite group of dogs for 5 days by oral gavage.

	Gender	Compound detected	C_max_ (ng/mL)		AUC_last_ (h^∗^ng/mL)		T_1/2_ (h)
			Day 1	Day 5	Day 1	Day 5	Day 5
EC3039 (1 mg/kg)	M	EC3039	400	2,300	2,490	72,100	33
		EC1728^1^	5	101	26	6,370	–
	F	EC3039	568	17,300	3,940	361,000	40
		EC1728	4	404	24	25,200	–
EC3039 (5 mg/kg)	M	EC3039	16,900	32,100	46,500	463,00	27
		EC1728	54	772	295	50,200	–
	F	EC3039	18,000	30,900	67,200	589,000	25
		EC1728	50	606	298	39,000	–
EC1728 (1 mg/kg)	M	EC1728	330	1,900	1,690	32,000	27
	F	EC1728	308	4,660	1,350	155,000	33
EC1728 (5 mg/kg)	M	EC1728	1,100	1,930	3,760	96,300	25
	F	EC1728	1,160	4,990	4,940	53,200	23

*EC3039 had improved PK properties compared to EC1728. EC3039 had higher systemic exposure (C_max_ and AUC) compared to EC1728, and exposure increased in a more than dose proportional manner for EC3039 and less than dose proportional manner for EC1728. Exposure for both compounds increased over the 5-days of dosing. ^1^EC1728 was detected in animals treated with EC3039, presumable from N-demethylation by CYP450; however, the T1/2 could not be calculated because it could not be fitted to a PK model.*

**FIGURE 3:**
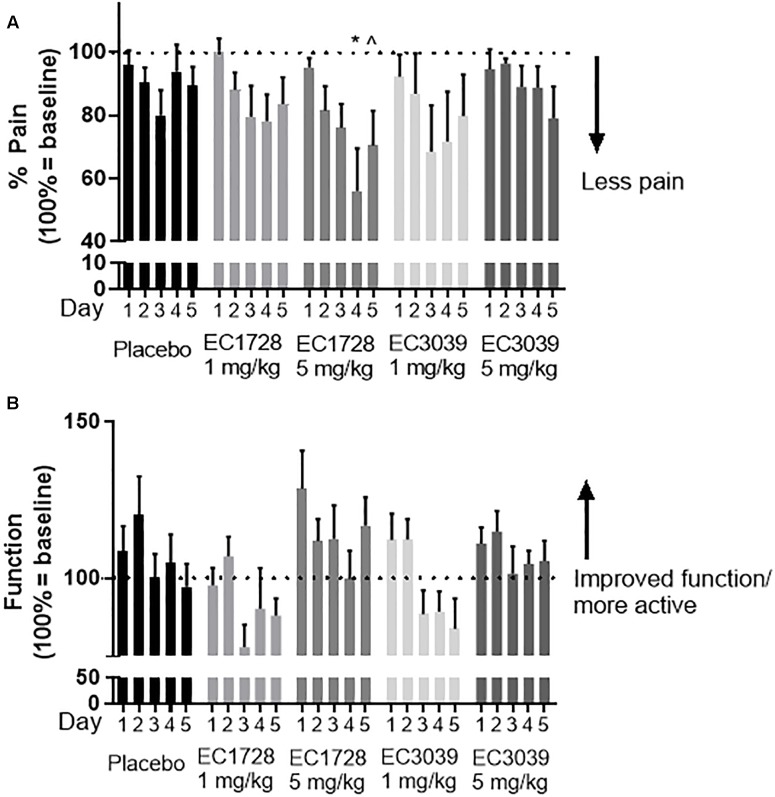
EC1728 at 5 mg/kg reduced pain and increased function in dogs with osteoarthritis (OA). In order to assess pain, technicians blinded to treatment answered a predetermined questionnaire based on observed dog behavior (on a scale of 0–2), and activity (on a scale of 0–5) of aged beagle dogs. Score 0 was classified as least pain and most function. The composite score was normalized for each individual dog to their predose score for pain **(A)** and function **(B)** are presented. **(A)** Dogs treated orally, once daily with EC1728 at 5 mg/kg for 5 days had significantly less pain compared to the placebo group. Placebo control consisted of administration of an empty 100% PEG300, and size 12 capsules from Torpac. Statistical significance was reached on day 4. After 5-days of dosing, EC1728 significantly reduced pain compared to vehicle control when analyzed by repeated measures ANOVA. There were no statistical differences identified for other treatment groups. **(B)** While no statistical significance was observed for improvement in function with any treatment group, dogs treated with EC1728 tended to have higher function scores than other treatment groups (*p* < 0.05 for treatment effect when analyzed by repeated measures ANOVA, but there was no statistically significant interaction between treatment groups). ^∗^*p* < 0.05 compared to placebo and ^∧^*p* < 0.05 for cummulative treatment effects when analyzed by repeated measure ANOVA.

**FIGURE 4:**
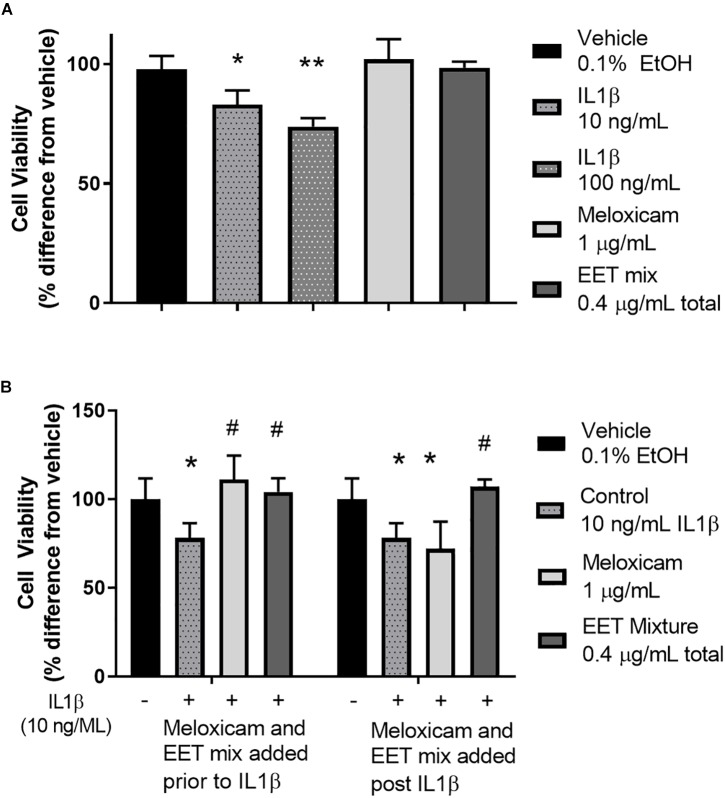
IL1β induced cytotoxicity was alleviated by meloxicam or EET mixture in an *in vitro* model of OA. **(A)** CnC cells treated 2 h with IL1β showed a dose dependent increase in cytotoxicity measured by Alamar Blue. The meloxicam or EET mixture, treated for 48 h, in the absence of IL1β, had no effect on healthy CnC cells. **(B)** Chondrocytes treated with IL1β for 2 h before or after treatment with meloxicam (1 μg/mL) or a mixture of EETs (5,6 EET, 8,9 EET, 11,12 EET, and 14,15 EET each at 0.1 μg/mL for a total EET concentration of 0.4 μg/mL). Meloxicam (added before the addition of 10 ng/mL of IL1β) or EET mixture, added either before or after a 2-h IL1β treatment significantly reduced IL1β induced cytotoxicity. Meloxicam added after IL1β did not result in significant reduction in cytotoxicity. ^∗^*p* < 0.05, ^∗∗^*p* < 0.005 compared to vehicle not treated with IL1β; #*p* < 0.005 compared to vehicle treated with IL1β.

### Efficacy

Dogs tolerated all treatments well, with no treatment related adverse events reported (a list of events recorded for the study can be found in Supplementary Table S4). Body weights were recorded on the day prior to dosing and again on day 5 of dosing. No dogs gained or lost more than 0.5 kg of body weight in any of the treatment groups. Dogs were assessed for pain and function based on a 12-question survey. The survey was administered for 5 days prior to dosing: the first 2 days were defined as an acclimation period and the final 3 days were averaged to provide baseline scores. One to two days prior to treatment, and after establishing baseline measurements, each dog was randomized based on age, radiograph score, and survey response. Prior to treatment, all groups averaged a score of 20 out of 24 for pain, and 14 out of 48 for function, except for the 1 mg/kg EC3039 group which averaged 15 for function. There were no significant differences between the baseline assessments of each group. One-way ANOVA analysis indicates that over the 5 days of treatment, EC1728 administered at 5 mg/kg significantly reduced pain in dogs with OA (Figure 3A). The maximum effect was observed on day 4 with an average of 44% reduction in pain compared to predose values. Vehicle treated animals were reported to have only a 7% reduction on this day. While there were no statistical differences between function scores for each of the treatment groups, dogs treated with EC1728 at 5 mg/kg tended to have increased function scores compared to the other groups (Figure 3B). After 5-days of dosing, dogs showed a 16% increase in function compared to a 3% decrease in function reported in the vehicle treated animals. Both pain and function scores showed high variability throughout the course of treatment. In addition, we did not observe significant association between pain and function in our data when analyzed by a non-parametric Spearman correlation coefficient.

Concentrations of EC3039 and EC1728 were assessed in the whole blood and synovial fluid of dogs in each treatment group (*n* = 8). No compounds were detected in the placebo treated group. Most animals had compound concentrations measured above the IC_50_ for enzyme inhibition in both blood and synovial fluid indicating that the compound was able to penetrate into the target tissue (Supplementary Table S5). Dogs treated with EC3039 had higher C_max_ and AUC concentrations of sEHI in their blood and synovial fluid than dogs treated with EC1728 (approximately 2× more in the low dose group and 10× more in the high dose group). Dogs treated with EC3039 also had detectable levels of EC1728 in their blood and synovial fluid, presumably due to demethylation of the urea. However, these concentrations were lower than dogs administered parent EC1728, indicating that EC3039 is acting largely as a direct sEHI, and EC3039 is a poor prodrug for EC1728. Of note, one dog (dog 2.4, Supplementary Table S5) treated with 5 mg/kg EC1728 had lower amounts of compound detected in the blood and synovial fluid (34 ng/mL in the blood on day 5 compared to 395 ng/mL average for the remainder of the group, and 80–95 ng/mL in the synovial fluid vs. 988 ng/mL averaged for the group on day 5). Due to low exposure levels, this dog was removed from analysis.

**FIGURE 5:**
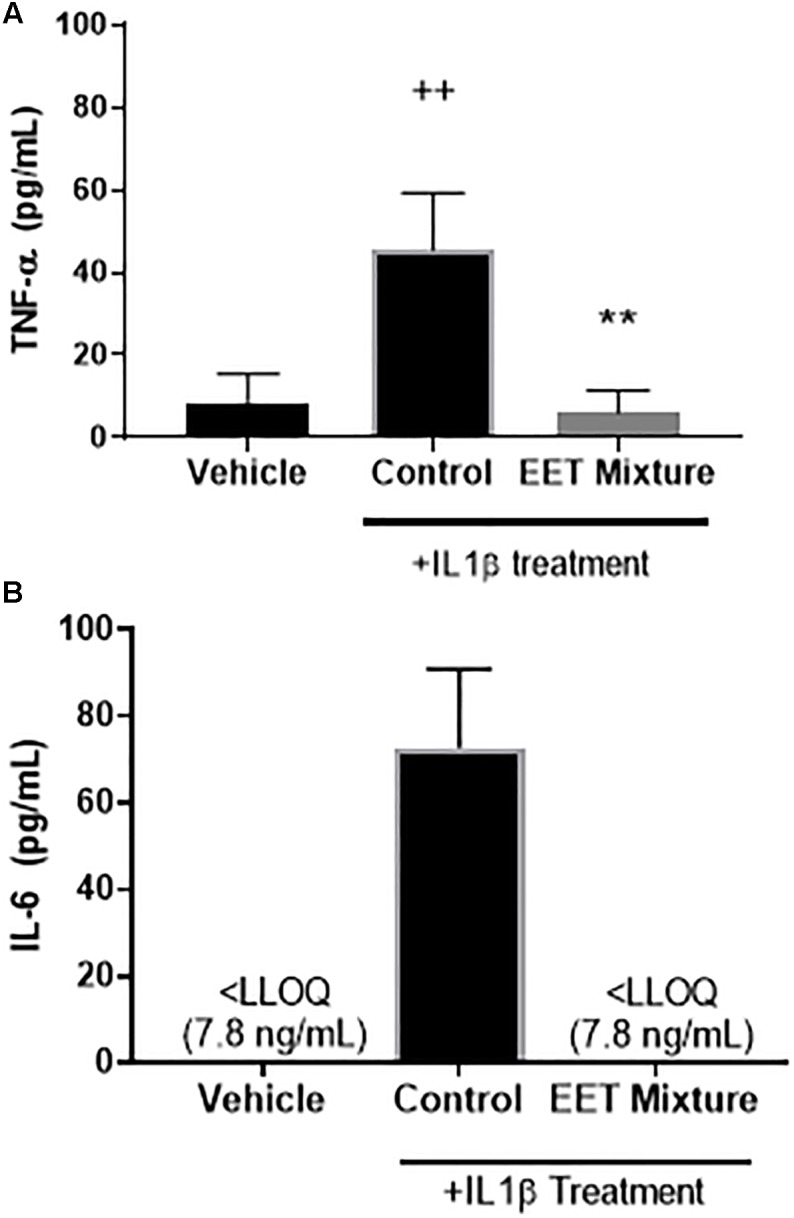
EET mixture (0.4 μg/mL) significantly reduced inflammatory cytokines, TNF-α and IL-6 after a 2-h treatment of IL1β (10 ng/mL). Inflammatory cytokines, TNF-α **(A)** and IL-6 **(B)**, protein levels were measured by ELISA 48-h after a 2-h treatment of IL1β or vehicle. Treatment with IL1β significantly increased both cytokines. When a mixture of EET regioisomers (0.1 μg/mL of each for a total of 0.4 μg/mL total) was added to the media after IL1β was removed, IL-6 and TNF-α decreased to levels comparable to cells not treated with IL1β. *p* < 0.005 between IL1β treated and not-treated (++) and post IL1β treated vehicle vs. EET (^∗∗^). There was no significant difference between cells not treated with IL1β and cells treated with EET mixture after a 2-h after IL1β.

### *In vitro* Model of Chondrocyte Cytotoxicity

To elucidate the role of increasing EET concentrations in arthritic animals, EET mixtures were evaluated for their ability to decrease inflammatory cytokines and protect chondrocytes against IL1β induced cytotoxicity. CnC cells treated with IL1β showed a significant and dose dependent increase in cytotoxicity (Figure 4A) that was eliminated when the chondrocytes were pretreated with either meloxicam or EETs, or treated post-IL1β with a mixture of EETs (Figure 4B). The *in vitro* model evaluated two concentrations of IL1β, 10, and 100 ng/mL, to simulate OA conditions in patients. A dose dependent reduction in cytotoxicity was observed (Figure 4A), and 10 ng/mL was selected as the concentration for continued experiments to better replicate *in vivo* conditions [0.288 ng/mL reported in the synovial fluid of humans with OA (McNulty et al., 2013)]. Interestingly, treating CnC cells with meloxicam after IL1β did not result in significant improvement in cytotoxicity. IL1β induces COX activity and an increase in PGs such as PGE2 (Attur et al., 1998); thus, it is no surprise that post treatment with a COX inhibitor would fail to attenuate inflammation in the presence of PGs (Hinson et al., 1996). Conversely, the anti-inflammatory action of EETs act downstream of PGE2 (Inceoglu et al., 2011); therefore, it is expected that EETs would work to counter PGE2 induced inflammation post IL1β treatment when the NSAID, meloxicam, did not. Protein measurement of inflammatory cytokines IL-6 and TNF-α were reduced in chondrocytes treated with a mixture of EETs (Figure 5); confirming that EETs protect chondrocytes through an anti-inflammatory response.

## Discussion

To assess the effects of sEHI on treating pain in animals with OA, arthritic dogs were treated orally with either of two sEHI (EC3039 or EC1728) for 5 days, and pain and function were measured compared to untreated placebo animals as a measure of efficacy. Although a potent inhibitor, EC1728 has poor solubility in both water and common organic solvents as well as a high melting point. EC3039 was synthesized to improve solubility without significantly altering the structure: by adding a methyl group to the urea pharmacophore, potency only slightly decreased but water solubility improved by almost 30-times and melting point was reduced from 240 to 92.4°C. Additions of N-methyl groups on the urea of sEHI are generally less active because the two hydrogens on the urea are needed to bond to the catalytic aspartic acid in the active site of the enzyme (Gomez et al., 2006). EC3039 was ∼5× less potent than EC1728 presumably due to the addition on the urea pharmacophore; although more active than most other compounds with N-methyl additions on the urea. Improved solubility and lower melting resulted in improved PK as evident by the increase C_max_ and AUC of EC3039 compared to EC1728, and it was hypothesized that improvements in PK would compensate for decreased potency. Despite the less than ideal physiochemical properties of EC1728, the compound displayed favorable PK, and both EC3039 and EC1728 had concentration measured in the plasma and synovial fluid well above the IC_50_ of sEH. Only EC1728 at 5 mg/kg was effective in reducing pain associated with OA in dogs. While a full PK profile was conducted in satellite animals, in order to not interfere with pain assessments, study animals only had synovial fluid collected on days 1 and 5 after pain assessments were complete. Because a full timecourse of distribution in the synovial fluid was not feasible without interfering with efficacy measurements, it is unknown if EC1728 remained in the synovium longer than EC3039. It is also possible that EC1728 is more effective in penetrating the cells and interacting with the target enzyme than EC3039. In addition to differences in potency and distribution, target occupancy, or amount of time the drug is bound to the enzyme, could also influence the efficacy of the compounds. Target occupancy is a predictor of efficacy, and although the exact resonance time of the drug on the enzyme is unknown, the decrease in potency suggests an increase in K_off_; thus, improved efficacy observed with EC1728 could be a function of potency and target occupancy (Tonge, 2018). Further investigation is needed to test this and understand the additional benefits provided by EC1728.

The sEHI work to inhibit the metabolism of beneficial, anti-inflammatory EpFA, thereby increasing their biological concentrations. In the *in vitro* cytotoxicity assay, EETs proved to be anti-inflammatory and decreased the inflammatory cytokines, IL-6 and TNF-α, after canine chondrocytes were incubated with IL1β. The primary effect is hypothesized to be independent of prostaglandin production since efficacy was achieved after IL1β incubation and reported stimulation of PG (Johnson et al., 2016). Furthermore, treatment with the NSAID, meloxicam, was only effective in protecting chondrocytes from IL1β induced cytotoxicity if added prior to IL1β stimulation (Figure 4B); however, in the longer term, EETs have been shown to down regulate induced COX-2 protein and message (Schmelzer et al., 2006a). Chondrocytes are cells in joint cartilage that produce cartilage and regulate cytokine release in response to stress stimuli, and decreased chondrocyte viability in response to injury or inflammatory stimuli has been associated with the progression of OA (Akkiraju and Nohe, 2015). IL1β has been associated with pathological changes in OA by increasing matrix metalloproteases and inflammatory cytokines such as IL-6 and TNF-α (Pelletier et al., 1995; Kapoor et al., 2011). Chondrocytes express the receptor for IL1β and are considered the main cellular target of this proinflammatory cytokine in cartilage (Daheshia and Yao, 2008). Thus, *in vitro* data predict that increased EET concentrations will reduce synovial inflammation *in vivo* and protect chondrocytes.

In addition to anti-inflammatory effects, EpFA also reduce ER-stress and are proposed targets for alleviating neuropathic pain (Inceoglu et al., 2017). Because OA is characterized by both inflammatory and neuropathic pain (Dimitroulas et al., 2014), a dual approach treating both inflammation, and neuropathic pain would improve patient outcomes especially considering that NSAIDs are ineffective in treating neuropathic pain (Rasmussen-Barr et al., 2016), and current neuropathic pain treatments (such as opioids and pentanoids) have serious addiction potential with debilitating and often deadly side-effects. Additionally, previously published studies demonstrated the beneficial effects of targeting dual inhibition of sEHI and COX. For example, in a mouse model of LPS, dual inhibition increased antinociception (Schmelzer et al., 2006b). In addition to synergistic efficacy, others have observed reduced ulcers in mice treated with both the NSAID, diclofenac, and sEHI compared to diclofenac treated mice alone (Goswami et al., 2016, 2017). Thus, safer and more effective treatment options are greatly needed, and the combination of sEHI and NSAID could provide a powerful approach to eliminate pain and co-morbidities of patients suffering from OA without deleterious side effects. Targeting OA from two pathways of decreasing PG production, through NSAIDs, as well with sEHI to reduce ER-stress and inflammation in the presence of PG could provide an effective dual approach to reduce inflammatory and neuropathic pain in OA patients.

Osteoarthritis is a debilitating disease for both humans and companion animals. The results of this study identified a lead compound to further investigate the use of sEHI to treat OA in companion animals. In addition, natural disease in dogs is often used as a more appropriate model for human disease than rodent models (Hoffman et al., 2018), providing evidence that sEHI is a potential target for human OA as well. A limitation of the study was the high variability observed in the *in vivo* study among each treatment group in the assessments of pain, function and PK, as well as the additional complications in using aged dogs where confounding factors could interfere with the interpretation of pain and mobility; although the limitations are somewhat offset by using a natural disease state in the animals to be treated. Variability is expected in naturally occurring disease, and as a proof of concept study, this study was designed to mimic treatment in a patient population. Future studies will identify mechanistic changes using both *in vitro* and *in vivo* models in rodents and companion animals to identify a detailed characterization of sEH activity in OA. The reduction in pain in the high dose of EC1728 provides justification for continued efforts in developing sEHI alone or in combination with NSAIDs for the treatment of OA in companion animals and humans.

## Ethics Statement

This study was conducted in accordance with InterVivo’s approved IACUC protocol, in compliance with the Animal Welfare Act (AWA) and Public Health Service Policy on Humane Care and Use of Laboratory Animals.

## Author Contributions

CBM, SH, KW, WS, and BDH conceived of the presented idea. SH synthesized the compounds and tested in these studies. CBM performed the *in vitro* cell experiments, microsomal stability, PK analysis, and analyzed the *in vivo* study results. KW and CM helped to supervise the project. JY and DW verified the analytical methods and analyzed the PK samples. DL oversaw the ELISA experiments. BDH provided overall supervision for the project. All authors discussed the results and contributed to the final manuscript.

## Conflict of Interest Statement

The University of California holds patents on the sEH inhibitors used in this study as well as their use to treat inflammation, inflammatory pain, and neuropathic pain. BDH and CM are cofounders and KW, JY, and WS are employees of EicOsis L.L.C., a startup company advancing sEH inhibitors as potential therapeutics. EicOsis provided funding for studies contracted to InterVivo. The remaining authors declare that the research was conducted in the absence of any commercial or financial relationships that could be construed as a potential conflict of interest. The reviewer DP declared a past co-authorship with several of the authors, SH, JY, BDH, to the handling Editor.
