# Supplementary material for: Pharmaceutical Effects of Inhibiting the Soluble Epoxide Hydrolase in Canine Osteoarthritis
Source: Front Pharmacol. 2019 May 31;10:533. doi: 10.3389/fphar.2019.00533 (PMC6554663; doi:10.3389/fphar.2019.00533)
Supplement: Supplementary file 1 [file Table_1.docx]

**Supplementary Materials**

Pharmaceutical Effects of Inhibiting the Soluble Epoxide Hydrolase in Canine Osteoarthritis

Cindy B. McReynolds^1,2,^ Sung Hee Hwang^1,2^, Jun Yang^1,2^, Debin Wan^1^, Karen Wagner^1,2^, Christophe Morisseau^1^, Dongyang Li^1^, William K. Schmidt^2^, Bruce Hammock*^1,2^

^1^ Department of Entomology and Nematology and UC Davis Comprehensive Cancer Center, University of California, Davis, USA.

^2^ EicOsis, Davis, CA, USA

*** Correspondence:**Dr. Bruce Hammock
bdhammock@ucdavis.edu

**S1. Chemical synthesis of EC3039**

EC1728 (***t*-TUCB**) was synthesized according to the synthetic procedure reported previously (Hwang, et al. 2007).

Preparation of *trans*-4-{4-[3-(4-trifluoromethoxy-phenyl)-ureido]-cyclohexyloxy}-benzoic acid (**A**).

To a solution of N-methyl-4-(trifluoromethoxy)aniline (1.91 g, 10 mmol) and 4-[(trans-4-aminocyclohexyl)oxy]-benzonitrile (2.16 g, 10 mmol) in THF (100 mL) was added triphosgene (1.04 g, 3.5 mol) at 0 ^o^C. The reaction mixture was warmed up to room temperature. After stirring for 2h, water was added, and the reaction mixture was extracted three times with EtOAc. The combined organic layers were dried with MgSO_4_ and the solvent was removed in vacuo. The remained crude solid was purified by column chromatography to obtain the titled compound (2.6 g, 60% yield). mp 104.0-106.9 ^o^C. ^1^H NMR (600 MHz, DMSO-*d*_6_) δ 7.73 (d, *J* = 7 Hz, 2H), 7.36 (d, *J* = 8 Hz, 2H), 7.32 (d, *J* = 8 Hz, 2H), 7.10 (d, *J* = 7 Hz, 2H), 6.13 (d, *J* = 8 Hz, 1H), 4.41-4.32 (m, 1H), 3.56-3.48 (m, 1H), 3.17 (s, 6H), 2.08-2.01 (m, 2H), 1.87-1.80 (m, 2H), 1.46-1.37 (m, 4H).

Preparation of *trans*-4-{4-[3-methyl-3-(4-trifluoromethoxy-phenyl)-ureido]-cyclohexyloxy}-benzoic acid (***t*-MTUCB**).

To a solution of the compound **A** (2.5 g, 5.8 mol) in EtOH (58 mL) was added 6N NaOH solution(33.8 mL) at room temperature. The reaction mixture was gently heated up to 90 ^o^C and stirred overnight. The reaction mixture was cooled to 0 ^o^C and acidified with conc. HCl. After evaporating ethanol, the precipitates were filtered and washed with water. The crude solid was purified by column chromatography to obtain the titled compound (2.3 g, 89% yield). mp 92.4-94.7 ^o^C. ^1^H NMR (400 MHz, DMSO-*d*_6_) δ 12.59 (s, 1H), 7.85 (d, *J* = 8.8 Hz, 2H), 7.37 (d, *J* = 9.1 Hz, 2H), 7.32 (d, *J* = 8.9 Hz, 2H), 7.00 (d, *J* = 8.8 Hz, 2H), 6.13 (d, *J* = 7.6 Hz, 1H), 4.39-4.28 (s, 1H), 3.59-3.47 (s, 1H), 3.17 (s, 3H), 2.11-1.99 (s, 2H), 1.89-1.77 (s, 2H), 1.50-1.33 (m, 4H). Anal. Calcd for C_22_H_23_F_3_N_2_O_5_: C, 58.41; H, 5.12; N, 6.19. Found: C, 58.35; H, 5.03; N, 6.09.

Preparation of *trans*-4-{4-[3-(4-trifluoromethoxy-phenyl)-ureido]-cyclohexyloxy}-benzoic-2,3,5,6-*d*_4_ acid (***t*-TUCB-*d*_4_**).

The compound was synthesized according to the synthetic procedure for *t*-TUCB using 4-fluorobenzonitrile-*d*_4_ instead of 4-fluorobenzonitrile (Hwang, et al. 2007). ^1^H NMR (600 MHz, DMSO-*d_6_*) δ 12.58 (s, 1H), 8.52 (s, 1H), 7.47 (d, *J =* 9 Hz, 2H), 7.22 (d, *J =* 9 Hz, 2H), 6.21 (d, *J =* 7 Hz, 1H), 4.49-4.42 (m, 1H), 3.57-3.48 (m, 1H), 2.10-2.02 (m, 2H), 1.96-1.90 (m, 2H), 1.54-1.32 (m, 4H).

**S2. LC/MS/MS method for PK analyses of 1728 and 3039**

The LC/MS/MS system consisting of Acquity UPLC system and Xevo TQS triple quadrupole system was used to measure the concentrations of inhibitors in the blood. The mobile phases of LC separations are 0.1% of formic acid (FA) in water (Phase A) and 0.1% of formic acid in acetonitrile (Phase B). The gradient of the separation is starting from 30% of phase B in the beginning to 50% of phase B at 2min then ramp to 95% at 2.2 min then came back to 30% of phase B at 3 min. 3 uL of the samples were injected on a 2.1 x 50mm Aquity UPLC BEH C18 1.7 um column. The mass spectrometer was operated under negative ESI mode using multiple reaction monitor (MRM) scan mode with the optimized conditions for the inhibitors. The detail transition parameters are listed in Table X. The conditions for the source were: capillary voltage at 3 kV, desolvation temperature at 300 C.

Table S1. The optimized parameters on Waters Xevo TQS system for measuring inhibitors.

| Analytes | MRM transition | Cone voltage (V) | Collison Energy (eV) |
| --- | --- | --- | --- |
| TAPU | 328.1 > 160.0 | 46 | 16 |
| CUDA | 339.3 > 214.2 | 22 | 38 |
| 1728 | 437.2 > 137.0 | 88 | 16 |
| 3039 | 451.2 > 260.1 | 56 | 16 |

**Supplementary Table S2:** Signalment for each dog enrolled in the study

| **DOG #** | **PERMANENT ID** | **AGE** | **SEX** | **TREATMENT**  **GROUP** | **RADIOGRAPHIC SCORE** |
| --- | --- | --- | --- | --- | --- |
| 1.1 | 0006E8F4E4 | 10.9 | M | EC1728 – 1mg/kg | 8 |
| 1.2 | 00074FF913 | 9.8 | F | EC1728 – 1mg/kg | 2 |
| 1.3 | 0006E94B2D | 11.2 | F | EC1728 – 1mg/kg | 3 |
| 1.4 | 00074F1D08 | 9.1 | F | EC1728 – 1mg/kg | 2 |
| 1.5 | 00064CA77C | 13.7 | M | EC1728 – 1mg/kg | 4 |
| 1.6 | 0006E935AA | 13.9 | F | EC1728 – 1mg/kg | 2 |
| 1.7 | 0006894B73 | 9.5 | F | EC1728 – 1mg/kg | 7 |
| 1.8 | 0006E96F82 | 12.8 | M | EC1728 – 1mg/kg | 1 |
| 2.1 | 00074F1660 | 8.7 | F | EC1728 – 5mg/kg | 7 |
| 2.2 | 00074F2D3A | 10.2 | F | EC1728 – 5mg/kg | 5 |
| 2.3 | 00074F1625 | 9 | F | EC1728 – 5mg/kg | 6 |
| 2.4 | 0006E8BCDC | 12.3 | M | EC1728 – 5mg/kg | 2 |
| 2.5 | 0006E95DFC | 11.3 | F | EC1728 – 5mg/kg | 10 |
| 2.6 | 0006E8EAEC | 12.3 | M | EC1728 – 5mg/kg | 3 |
| 2.7 | 00074EF48A | 9.5 | F | EC1728 – 5mg/kg | 12 |
| 2.8 | 00064DAD86 | 13.9 | M | EC1728 – 5mg/kg | 11 |
| 3.1 | 00071928DC | 9.8 | M | EC3039 – 1mg/kg | 7 |
| 3.2 | 00064D4AC8 | 13.7 | M | EC3039 – 1mg/kg | 1 |
| 3.3 | 00064CF01D | 14.5 | M | EC3039 – 1mg/kg | 14 |
| 3.4 | 00074EEABD | 7.9 | F | EC3039 – 1mg/kg | 2 |
| 3.5 | 00074FCAF5 | 9.8 | F | EC3039 – 1mg/kg | 18 |
| 3.6 | 00064DC04B | 10.7 | F | EC3039 – 1mg/kg | 8 |
| 3.7 | 0006E8885A | 11.4 | F | EC3039 – 1mg/kg | 9 |
| 3.8 | 00074FFFF7 | 9.8 | F | EC3039 – 1mg/kg | 9 |
| 4.1 | 0006E8EFF1 | 10.2 | F | EC3039 – 5mg/kg | 5 |
| 4.2 | 0006E96E9B | 13.3 | F | EC3039 – 5mg/kg | 2 |
| 4.3 | 0006E8CF7A | 12.9 | M | EC3039 – 5mg/kg | 4 |
| 4.4 | 00074FC5B5 | 7.8 | F | EC3039 – 5mg/kg | 6 |
| 4.5 | 00064E0B44 | 12.3 | F | EC3039 – 5mg/kg | 15 |
| 4.6 | 00064E0532 | 12.3 | F | EC3039 – 5mg/kg | 16 |
| 4.7 | 0006E91BD9 | 13.6 | M | EC3039 – 5mg/kg | 7 |
| 4.8 | 00074FFBF9 | 12.4 | M | EC3039 – 5mg/kg | 1 |

| **Supplementary Table S3:** Pain and Function Questionnaire adapted from the Canine Brief Pain Inventory (Brown, 2007)  Pain was scored from 0 (worst pain) to 2 (least pain)  Function was scored from 0 (with great ease) to 5 (with great difficulty) |
| --- |
| 1. Ease of rising / lying down if observed? 2. General Alertness? (not included in composite score) 3. Ease of initiating walking? 4. General Activity? 5. Willingness to Walk? 6. Willingness to Trot? 7. Willingness to Gallop? 8. Willingness to Step / Jump Over Low Obstacles? 9. Willingness to Climb Stairs? 10. Willingness to Descend Stairs? 11. Willingness to Rear / Jump for Food? 12. Willingness to Jump Down from Step / Perch? 13. Overall Impressions (not included in composite score) 14. Overall Pain assessment (scored 0-4) |

**Supplementary Table S4:** Adverse events

A summary of health cases filed over the course of the study can be found below. There was no evidence of treatment-specific adverse events in any group; however, the findings for dog 1.2 (1 mg/kg EC1728) and dog 3.6 (1 mg/kg EC3039) may have impacted the data on days the adverse events were ongoing. However, it is unlikely that this would have substantially impacted the current conclusions.

*Table 2: Summary of health cases*

| **Date Noted** | **Study Phase Noted** | **ID** | **Description** | **Findings on Examination** | **Treatment** | **Treatment Group / Related** |
| --- | --- | --- | --- | --- | --- | --- |
| N/A | Pre-Study | 1.7 | Pre-existing condition of glaucoma | N/A | Azopt TID and Maxidex BID (ongoing) | EC1728 – 1mg/kg / Not Related |
| 2015-09-23 | Baseline | Satellite-1F | Yellow discharge right eye | 2015-09-23: Mucoid discharge from right eye, minor abrasion beside eye  2015-09-24: Normal tearing, no swelling or discharge from abrasion. Normal. | 2015-09-23: Flushed with eye stream. Reassess in 24-48 hours | EC1728 – 1mg/kg / Not Related |
| N/A | Pre-Study | 1.7 | Pre-existing condition of allergic dermatitis | N/A | Diphenhydramine 25mg TID (ongoing) | EC1728 – 1mg/kg / Not Related |
| 2015-09-27 | Baseline | 1.2 | Non weight bearing on left hind | 2015-09-28: Fully weight bearing. Animal normal | None. | EC1728 – 1mg/kg / Not Related |
| N/A | Pre-Study | 2.8 | Pre-existing condition of laceration on left hind | N/A | Clindamycin 150mg BID 2015-09-20 to 2015-10-05 | EC1728 – 5mg/kg / Not Related |
| 2015-09-28 | Treatment  (Day 0) | Satellite-3M | 3cm multi nodular mass on inner left mandibular lip | 2015-09-28: Firm mass. Multilobulated, irregular, 3cm, pedunculated, on buccal surface mucosa at commissure of upper/lower lip | Remove surgically once study is completed. | EC3039 – 1mg/kg / Not Related |
| 2015-10-02 | Treatment  (Day 4) | 3.6 | Raw spot on left hip | 2015-10-02: Moist dermatitis | Hibitane ointment and chlorhexidine soak BID  2015-10-02 to 2015-10-07 | EC3039 – 1mg/kg / Unlikely Related |
| 2015-10-06 | Treatment  (Day 8) | Satellite- 4M | Moist dermatitis of ventral neck (suspected clipper burn) | N/A | None started prior to study completion | EC3039 – 5mg/kg / Not Related |

**Supplementary Table S5. EC3039 and EC1728 exposure in the synovial fluid was measured at concentrations above the IC_50_.**

Dogs had higher concentration of sEHI in the synovial fluid compared to concentration in the blood and exceeded the IC_50_ for each compound (EC_50_ of EC3039 = 2 ng/mL and the IC_50_ of EC1728 is 0.4 ng/mL). There was no statistically significant correlation between drug levels and pain or function scores.

1. Summary table of results: synovial fluid and blood concentrations of sEHI in dogs administered EC1728

| **1 mg/kg, po, qd x 5** | | | | **5 mg/kg, po, qd x 5** | | | |
| --- | --- | --- | --- | --- | --- | --- | --- |
| **Compound detected:**  **EC1728 (ng/mL)** | | | | **Compound detected:**  **EC1728 (ng/mL)** | | | |
|  | **Synovial** | | **Blood** |  | **Synovial** | | **Blood** |
| **Dog** | **D1** | **D5** | **D5** | **Dog** | **D1** | **D5** | **D5** |
| 1.1 | 483 | 315 | 82 | 2.1 | 1470 | 672 | 228 |
| 1.2 | 111 | 171 | 40 | 2.2 | 460 | 668 | 493 |
| 1.3 | 384 | 214 | 36 | 2.3 | nc | 2170 | 244 |
| 1.4 | 917 | 945 | 171 | 2.4 | 95 | 80 | 34 |
| 1.5 | 331 | 239 | 31 | 2.5 | 2270 | 1000 | 206 |
| 1.6 | 206 | 206 | 72 | 2.6 | 301 | 1150 | 348 |
| 1.7 | 455 | 378 | 76 | 2.7 | 1180 | 833 | 314 |
| 1.8 | 232 | 328 | 64 | 2.8 | 2220 | 1330 | 935 |

nc: not collected

1. Summary table of results: synovial fluid and blood concentrations of sEHI in dogs administered EC3039

| **1 mg/kg, po, qd x 5** | | | | | | | **5 mg/kg, po, qd x 5** | | | | | | |
| --- | --- | --- | --- | --- | --- | --- | --- | --- | --- | --- | --- | --- | --- |
|  | **Compound detected:**  **EC3039 (ng/mL)** | | | **Compound detected:**  **EC1728 (ng/mL)** | | |  | **Compound detected:**  **EC3039 (ng/mL)** | | | **Compound detected:**  **EC1728 (ng/mL)** | | |
|  | **Synovial** | | **Blood** | **Synovial** | | **Blood** |  | **Synovial** | | **Blood** | **Synovial** | | **Blood** |
| **Dog** | **D1** | **D5** | **D5** | **D1** | **D5** | **D5** | **Dog** | **D1** | **D5** | **D5** | **D1** | **D5** | **D5** |
| 3.1 | 3100 | 1440 | 346 | 100 | 87 | 12 | 4.1 | 5220 | 16100 | 722 | 259 | 320 | 38 |
| 3.2 | 1390 | 586 | 81 | 77 | 69 | 9 | 4.2 | 14300 | 0 | 6190 | 458 | 50 | 100 |
| 3.3 | 3430 | 2190 | 408 | 123 | 111 | 23 | 4.3 | 14700 | 11700 | 5650 | 481 | 720 | 79 |
| 3.4 | 2100 | 1930 | 328 | 65 | 100 | 13 | 4.4 | 9520 | 5960 | 929 | 704 | 464 | 115 |
| 3.5 | 1220 | 1630 | 126 | 111 | 111 | 17 | 4.5 | 5850 | 5830 | 859 | 269 | 208 | 62 |
| 3.6 | 2710 | 1480 | 176 | 143 | 80 | 16 | 4.6 | 13570 | 4640 | 672 | 565 | 318 | 71 |
| 3.7 | 4100 | 11900 | 5340 | 95 | 167 | 17 | 4.7 | 14290 | 9960 | 5550 | 250 | 217 | 33 |
| 3.8 | 3350 | 1840 | 347 | 115 | 94 | 12 | 4.8 | 14240 | 12400 | 6250 | 400 | 360 | 75 |

**References**

Hwang, S.H., Tsai, H.J., Liu, J.Y., Morisseau, C., and Hammock, B.D. (2007). Orally bioavailable potent soluble epoxide hydrolase inhibitors. J Med Chem 50(16), 3825-3840. doi: 10.1021/jm070270t.
